# Personality and Information Gathering in Free-Ranging Great Tits

**DOI:** 10.1371/journal.pone.0054199

**Published:** 2013-02-01

**Authors:** Thijs van Overveld, Erik Matthysen

**Affiliations:** Evolutionary Ecology Group, Department of Biology, University of Antwerp, Antwerp, Belgium; CNRS, Université de Bourgogne, France

## Abstract

One aspect of animal personality that has been well described in captivity, but received only little attention in studies in the wild, is that personality types may vary in their behavioural flexibility towards environmental changes. A fundamental factor underlying such differences is believed to be the degree to which individual behavior is guided by environmental stimuli. We tested this hypothesis in the wild using free-ranging great tits. Personality variation was quantified using exploratory behaviour in a novel environment, which has previously been shown to be repeatable and correlated with other behaviours in this and other populations of the same species. By temporarily removing food at feeding stations we examined whether birds with different personality differed in returning to visit empty feeders as this may provide information on how birds continue to sample their environment after a sudden change in conditions. In two summer experiments, we found that fast-exploring juveniles visited empty feeders less often compared to slow-exploring juveniles. In winter, sampling behaviour was sex dependent but not related to personality. In both seasons, we found that birds who sampled empty feeders more often were more likely to rediscover food after we again re-baited the feeding stations, but there was no effect of personality. Our results show that personality types may indeed differ in ways of collecting environmental information, which is consistent with the view of personalities as different styles of coping with environmental changes. The adaptive value of these alternative behavioural tactics, however, needs to be further explored.

## Introduction

The concept of animal personality or temperament (i.e., consistent and correlated individual differences in behaviour) is generally used to describe the predictable manner in which individuals respond to challenging or novel situations. These alternative response patterns allow individuals to be quantified along behavioural axes of aggressiveness, social tolerance, boldness or novelty seeking [1] with individuals on the extremes of these axes often being categorized as behavioural phenotypes having more proactive (e.g. aggressive, bold) or reactive (e.g. docile, risk-averse) coping strategies [2], [3]. In the last decade it has become clear that the existence of variation in personality types has far-reaching ecological and evolutionary implications [4]. For example, aspects of personality have been found to be heritable [5], [6], [7], [8], to be associated with fitness variation [9], [10] and to be a predictor of key processes in population dynamics such as dispersal and colonization success [11]. Furthermore, a growing number of studies have recently shown personality traits, particularly exploratory behaviour, to be associated with a wide range of ecological relevant behaviours including patterns of space use [12], [13], [14], [15] and/or foraging [16], [17], [18]. However, how personality differences measured in captivity can predict such patterns in the wild still remains poorly understood.

One behavioural mechanism that has been proposed, among others, is that personality types may vary in the capacity to adjust their behaviour to changes in environmental conditions [2], [19], [20]. A main factor underlying such differences in behavioural flexibility is believed to be the degree to which behaviour is guided by stimuli from the environment [19], [21]. Whereas some individuals rely on detailed information available in their environment and readily adjust their behaviour to a new situation, others are more driven by internal stimuli (i.e. previous experience) and tend to behave more rigid or routine-like. Such differences in the extent to which individuals perceive and use environmental information are considered to be a fundamental aspect of personality variation and have been well described in studies in captivity. In rodents for example, maze experiments showed that proactive individuals paid less attention to changes in maze structure, whereas reactive individuals responded by re-exploring their environment [22]. More recently, behavioural flexibility has also been tested in captive fish and birds using food relocation experiments, which showed that reactive individuals found new food locations relatively quickly after food removal, while proactive individuals initially kept returning to the original location of the food resource [23], [24], [25]. Differences in flexibility of behaviour have been further demonstrated in various other situations and studies have confirmed that in reactive individuals there is a stronger stimulus-response relationship compared to proactive individuals [2], [19], [21].

While behavioural flexibility in relation to personality variation is a well-established concept in studies on animals in captivity, little is still known about how such differences are expressed in the wild. This is surprising given that personality differences in behavioural flexibility and information use may potentially be of great importance in explaining variation in ecologically-relevant behaviours such as space use and foraging [20]. For example, in a recently conducted field experiment, we tested for personality differences in behavioural flexibility in great tits by looking at the spatial response to a change in food supply, and relating this to exploratory behaviour in captivity. We showed that slow-exploring (i.e. reactive) individuals only gradually shifted their home-range, whereas fast-exploring (i.e. proactive) individuals quickly moved to other foraging areas further away [14], indicating that fast and slow explorers differ in the way they cope with sudden uncertainties in food conditions. However, it is an open question whether this result should be interpreted in terms of higher or lower flexibility in behaviour, because both fast and slow personality types changed their spatial behaviour over time.

Here we present data on the same food-manipulation experiment mentioned above combined with an additional experiment performed in winter where we investigate in more detail how birds respond to the sudden disappearance of food in terms of collecting and updating information, and how this relates to personality differences. In particular, we examined whether birds with different personality differed in returning to visit the empty feeders as this may provide information on how birds continue to sample their environment after a sudden change in conditions. We hypothesized that individuals who visit empty feeders more frequently may in general invest more time and energy in knowledge on local feeding opportunities and as a consequence may be better able to respond to environmental changes. Based on previous observations in the laboratory we expected slow explorers to sample empty feeders more often because they tend to rely more on detailed information in their environment (i.e., their behaviour is driven by external stimuli), while we expected fast explorers to sample less and faster because they behave more routine-like (i.e., their behaviour is internally driven). In addition, if sampling reflects an adaptive strategy to cope with environmental variation in food availability, we expected sampling rates to be positively related to the rediscovery of re-baited feeders and slow explorers to rediscover food sooner than fast explorers. We carried out the experiments in different seasons because in summer the feeders were mainly visited by juveniles, while in winter the feeders were also extensively used by adults. This allowed us to examine whether the expression of personality differences in sampling behaviour may be more pronounced in birds that have less prior information about their environment (juveniles).

## Materials and Methods

### General Field Methods

**Figure 1:**
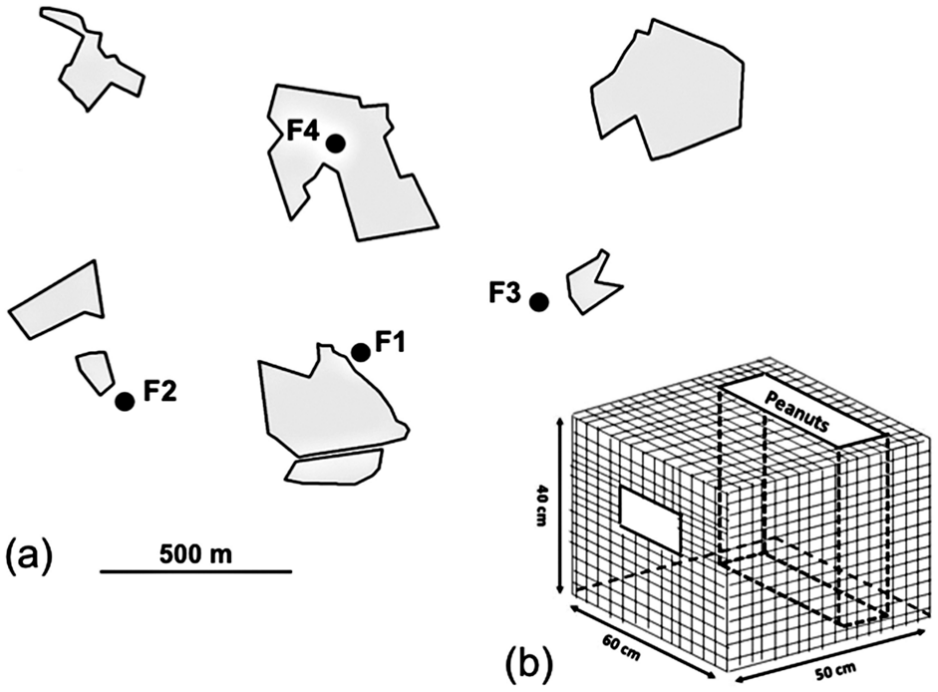
Locations of feedings station and schematic representation of feeding cage. Overview of experimental set-up: (a) locations of the four feeding stations in the central part of the study area. Each feeding station consisted of a table on which we placed a feeding cage (b) made out of wire mesh (1.5 cm * 1.5 cm) with a solid floor and containing a large peanut reservoir (dashed line). Birds could enter the cage only through a single opening (8 cm * 5 cm) on which we placed a registration antenna for reading pit tags implanted in great tits. The antenna was connected to a stationary decoder underneath the feeding table.

The study was conducted in the summer of 2007 and 2008 (July-August-September) and in the winter of 2008 (January–February) in a study area with scattered woodland fragments called ‘Boshoek’ in northern Belgium (51°80′80 N, 4°83′20 E). This area of approximately 10 km^2^ consists of 17 woodlots of mature managed forest ranging in size from 1 to 12 ha. Neighbouring woodlots are 100 to 600 m apart and separated by small residential areas and agricultural land. In spring 2007 we established four feeding stations (F1–F4) in the central part of the study area at approximately 500 m distance from each other (fig. 1a). Three feeding stations were located at the edge of woodland fragments of which F1 was situated in a garden, F2 in young deciduous scrub and F3 in a patch of willow trees. Feeding station F4 was located in the middle of a stand of beech trees. Feeding stations consisted of a wire cage (40 cm * 50 cm * 60 cm) containing a large peanut reservoir (Fig. 1b) that was mounted on a platform 1,5 m above the ground. Birds could enter the cage only through a single opening which during the experiments was surrounded by a registration antenna to record visiting rates (see below). In summer we baited the feeding tables from around the 20^th^ of June when the majority of fledglings in the population had reached independence. Birds visiting the feeders were trapped using mistnets from the beginning of July until the first week of August. In winter we baited the feeding stations around mid-December and started trapping birds from the beginning of January until the first week of February. We again used mistnets to trap birds visiting the feeding stations, but we also performed additional captures in the evening while birds were roosting in nestboxes. Birds not banded on their first capture in our study population were considered immigrants. Standard measurements (weight, tarsus length) were taken on every capture as well as a feather sample (i.e. 5–10 body feathers from the flank of the birds) in case of juvenile birds. We used molecular markers to sex juvenile birds caught in July and August [26], whereby DNA was isolated from feather quills [27]. To sex full-grown birds we used plumage characteristics [28]. Birds were aged as first year or older according to Jenni & Winkler [28].

### Novel Environment test

Birds captured in the field were transported to the lab within one hour (mistnet) or two hours (roosting birds in the evening). Distance between the study area and laboratory is approximately 10 km and birds were transported by car in a wooden box consisting of 12 separate compartments (15 cm *10 cm*8 cm), with a single bird in each compartment. In the laboratory, birds were housed individually under natural daylight regime in cages (0.8 m * 0.4 m * 0.5 m) with a solid bottom and top. Birds had *ad libitum* access to food (mealworms and peanuts) and water, and human disturbance was kept to a minimum. The exploratory behaviour of birds was measured on the following morning between 0900 and 1200 hours, using a novel environment test exactly as described in Dingemanse et al. [5]. Briefly, each bird was entered separately into a sealed room (4.0×2.4×2.3 m) containing five artificial trees and during the following 2 min, all movements among the different artificial trees (flights) and among the branches of individual trees (hops) were counted, including movements towards other structures such as lamps or sliding doors or to the floor, but not including movements on a single branch. The total number of movements was used as a measure of exploratory behaviour. After the test birds were caught and returned to their cages. All birds were released near their site of capture in the afternoon, but within 24 h after capture. Because exploration scores increase from summer to the start of the breeding season, we corrected the exploration scores for this seasonal trend using the equation: ‘season corrected exploration score’  =  ‘measured exploration score’ – (0.030× ‘July date’) +10, where ‘July date’ was the number of days from 1 July onwards [5], [29]. Our exploration score has significant repeatability (r = 0.42, n = 224) which is highly comparable to other studies of the same species [29].

### PIT-tags and registration system

After the birds were tested on their exploratory behaviour each individual was fitted with a Passive-Integrated Transponders (‘PIT’-tag: 11.5 mm * 2.1 mm, <0.1 g, Trovan ID100; Trovan, Ltd., Douglas, UK). PIT-tags are micro-chips which send a unique code when activated by a low-frequency magnetic field. We implanted the tags subcutaneously in the back of the bird, in the featherless area above the scapula on the left side. Procedures were similar as described in Nicolaus et al. [30]. To record the birds' visits at the feeding stations, we placed a flat metal rectangular loop antenna (8 cm * 5 cm) around the opening of the feeding cage (Figure 1b). This antenna generates an electromagnetic field within the loop for activating the PIT-tags and was connected to a stationary decoder (model LID665) with a memory capacity of 3200 recordings. The registration system was powered by a 12 Volt battery, which was replaced every three days.

### Ethics statement

We found no evidence for adverse effects of the experimental procedures. First, the average weight loss of the birds between arrival and departure of the laboratory was within their natural range (1.39 g ±0.68 SD, N = 242, [31]). Second, 220 of the 278 birds that were brought to the lab were later recorded at the feeders or trapped within the study area (79%). This percentage is within the range of the annual mortality rate of ca. 0.5 for this species [32] and comparable with recapture rates reported in other studies using similar procedures [16], [30], [33]. Our study complies with legal requirements for research in Belgium. Permission for capture, transport and short-term housing of great tits was granted by the Belgian Ringing Scheme and the Flemish administration (Agentschap voor Natuur en Bos).

### Food manipulation protocol

Summer experiments were performed at feeding stations F2 and F3 (fig. 1.). In both 2007 and 2008 we started to continuously record visits by PIT-tagged birds seven days prior to the food removal manipulation. Food was subsequently removed from two experimental feeders but not synchronously. Experimental feeders were emptied for 8 days and recordings lasted for at least seven days after we again re-baited the feeding station (see table 1 for an overview). In winter we used feeding stations F1, F2 and F4. Due to technical problems we only had continuous recordings for the seven days prior to the food removal for feeding station F4. For feeding station F1 we had pre-manipulation recordings for days 5, 3, 2 and 1 and for feeding station F2 for days 10, 9, 6, 5, 3, 2 and 1 (4 and 7 days respectively). In winter we first emptied the feeding station F1 followed by the simultaneous emptying of feeding stations F2 and F4. Re-baiting in winter occurred after four days instead of eight days (see table 1). We shortened this period because of the presence of many other (artificial) food resources in people's gardens surrounding the experimental sites and we wanted to avoid that only few birds would return to the feeding stations. After re-baiting the feeding stations we recorded birds' visits over at least 5 days, which was shorter than planned again due to technical problems. In both summer and winter emptying and re-baiting feeders always occurred in the evening while birds were roosting.

**Table 1:** Overview of experiments.

	Summer 2007		Winter 2008			Summer 2008	
Feeding stations	F2	F3	F1	F2	F4	F2	F3
Start registrations	13/aug	17/aug	16/feb	17/feb	20/feb	15/aug	11/aug
Emptying feeder	20/aug	24/aug	21/feb	27/feb	27/feb	22/aug	18/aug
Re-baiting feeder	28/aug	1/sep	25/feb	2/mar	2/mar	30/aug	26/aug
End registrations	4/sep	7/sep	6/mar	6/mar	6/mar	18/sep	18/sep

### Quantifying visiting and sampling rates

In this paper we refer to visiting behaviour when birds were recorded at the feeding station in the presence of food and to sampling behaviour when they were recorded in the absence of food. Because birds never flew directly into the cage but typically spent some time near the entrance before entering, we had many registrations per visit (>200.000 readings in total). To quantify visiting and sampling rates we therefore arbitrarily defined a visit as a series of registrations separated by an arbitrary cut-off time. The use of different cut-off times (5, 10, 15 or 20 minutes) resulted in different time intervals between visits and time spent at the feeder (i.e. the time between first and last registration of the same visit, see table 2), but individual visiting and sampling rates quantified by these different cut-off times were all highly intercorrelated (Pearson correlation; summer: visits r>0.88, sampling events r>0.91; winter: visits r>0.83, sampling r>0.96). In this paper we only present data based on a cut-off time of 10 minutes. Re-running all the analyses using visiting and sampling rates based on different cut-off times gave qualitatively similar results (details not shown).

**Table 2:** Median time between visits (interval) and time present at feeders (presence) based on series of registrations separated by different cut-off times (time between first and last registration; see methods).

		visits			sampling events (first day)		
	cut-off time	interval (min)	presence (sec)	N	interval (min)	presence (sec)	N
Summer							
	5 min	33	104	12114	70	21	749
	10 min	36	125	11302	79	26	678
	15 min	38	137	10284	86	37	632
	20 min	42	161	9253	90	44	591
Winter							
	5 min	31	131	4874	41	15	270
	10 min	36	169	4278	54	18	257
	15 min	41	208	3818	58	20	235
	20 min	47	247	3402	60	22	225

### Data composition

We tagged a total of 278 individuals over the three experimental periods (see table 1 for an overview on sample sizes). The number of tagged individuals was about equal at each site within each experimental period. Sex-ratios were close to 1∶1 at each site in all three periods. Birds tagged and assayed on exploration behaviour in summer were all juveniles, whereas in winter half of the birds were adults. Detection rate (percentage of released birds that visited at least one feeding station) was high in summer with at least one registration record prior to the food removal for 80% of the tagged birds (72% in 2007 and 92% in 2008). In winter, detection rate was lower with only 58% of the tagged birds being recorded before we removed the food. In all three periods there was no bias in detection rate after tagging towards sex, age or exploration score (Logistic regression p>0.3). In summer 2008, 21 adults tagged in winter were also detected at feeders, but never caught with mistnets. Given that these birds visited the feeders very infrequently both before and after food removal we excluded them from the analyses. Very few birds visited multiple feeders before the experiment (n = 10, only in winter). We did not exclude these individuals, because other birds could have done the same before we started the pit-tag registrations. For 10 juveniles we were unable to determine the sex.

### Statistical Analyses

**Figure 2:**
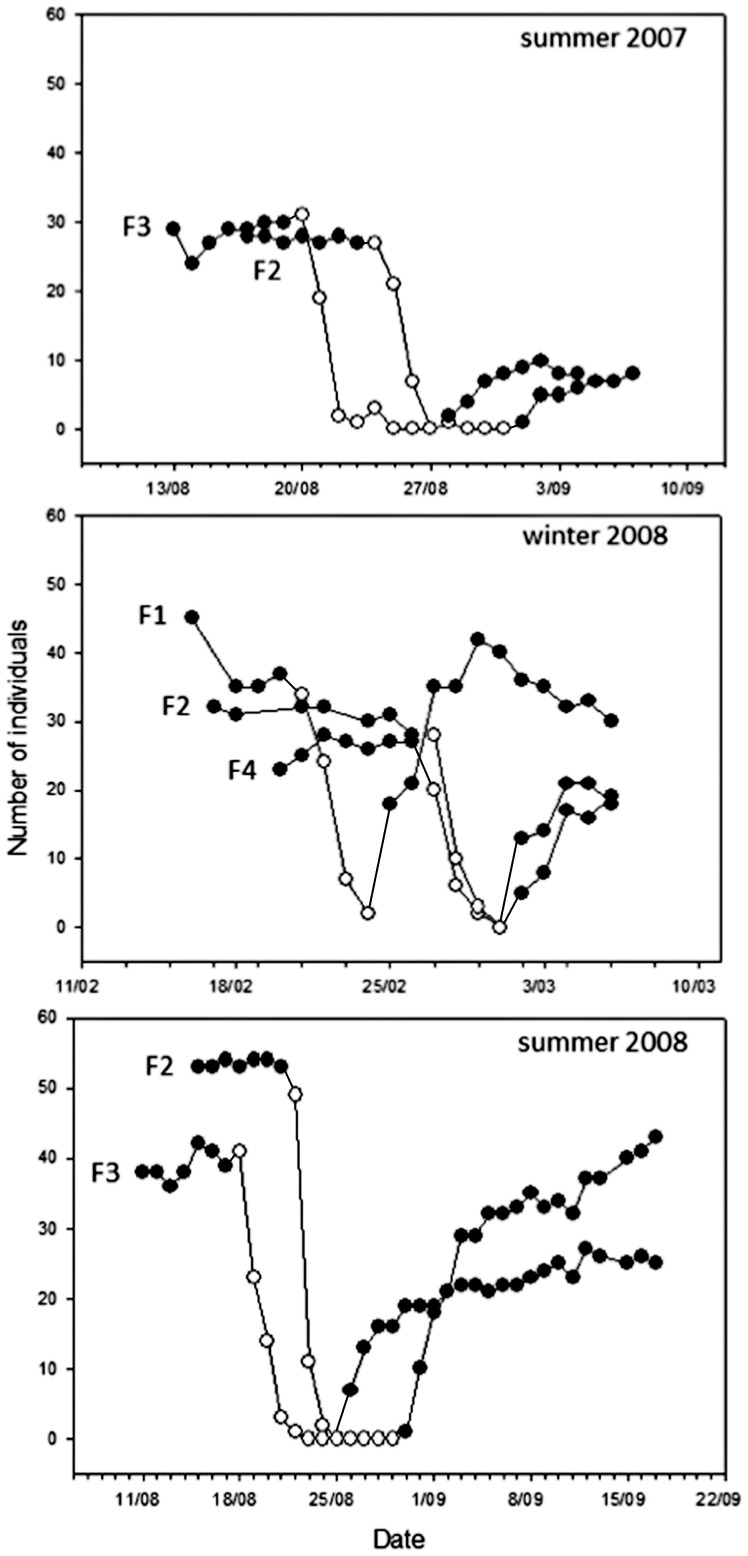
Number of individuals visiting the feeders before, during and after food removal. Number of individuals recorded per day at the feeding stations during the whole experimental period. Closed dots represent the number of individuals in the presence of food (before food removal and after re-baiting the feeding stations) and open dots the number of individuals after we removed the food.

All analyses were performed using SAS 9.2 software. Because of differences in day length between seasons we performed separate analyses on winter and summer data (summer 2007 and 2008 pooled). Birds may adjust their daily visiting and sampling rates according to the number of hours available for foraging for which we believe cannot easily be corrected for. Factors affecting daily visiting and sampling rates were analysed using mixed models (PROC MIXED in SAS) with ID as a random effect and Satterthwaite correction for the *df* [34]. Significance of random effects is reported based on likelihood ratio tests (LRT). Sampling rates in winter were log-transformed to reach normality of residuals (Shapiro – Wilk test, all other W>0.98). To test for factors explaining the rediscovery of food after re-baiting the feeding stations, we used Cox proportional hazards models [35].This model estimates the daily probability (or hazard) that an individual will return to the feeder for the first time. Main effects included in all models were exploration score, dispersal status (locally born individuals vs. individuals from outside the study population), sex, age (1^st^ year or older, winter only), body condition (residuals of body mass over tarsus), year, and feeder location. Dispersal status (locally born individuals vs. immigrants) was included because prior experience with the area may affect the birds' spatial behaviour [14] and is itself correlated with exploration score [13], [36]. In models on visiting rates we included date as a covariate to correct for seasonal variation in visiting rates. In models on sampling rates we included days since food removal as a covariate because sampling occurred primarily on the first day of the food removal and strongly decreased afterwards (Figure 2). We also included the interaction term exploratory behaviour * days since food removal to test whether relationships between exploratory behaviour and sampling rates changed over time. Because the rate of visits before food removal was related to the rate of sampling after food removal (Pearson correlation, r>0.37, p<0.001, for both summer and winter) we included the average visits per day as a covariate in models on sampling rates. We included capture method (roosting in nestboxes yes/no) as a covariate to take into account possible effects of territoriality on visiting and sampling rates in winter. In models on the rediscovery of food we included the total number of sampling events per individual as a covariate to test whether birds used their previously collected information. Model selection was based on a priori chosen fixed effects irrespective of their significance. All two-way interaction between exploration behaviour and other main effects were tested, but removed when non-significant.

## Results

### Pre- and post-manipulation visiting and sampling rates

#### Summer

**Figure 3:**
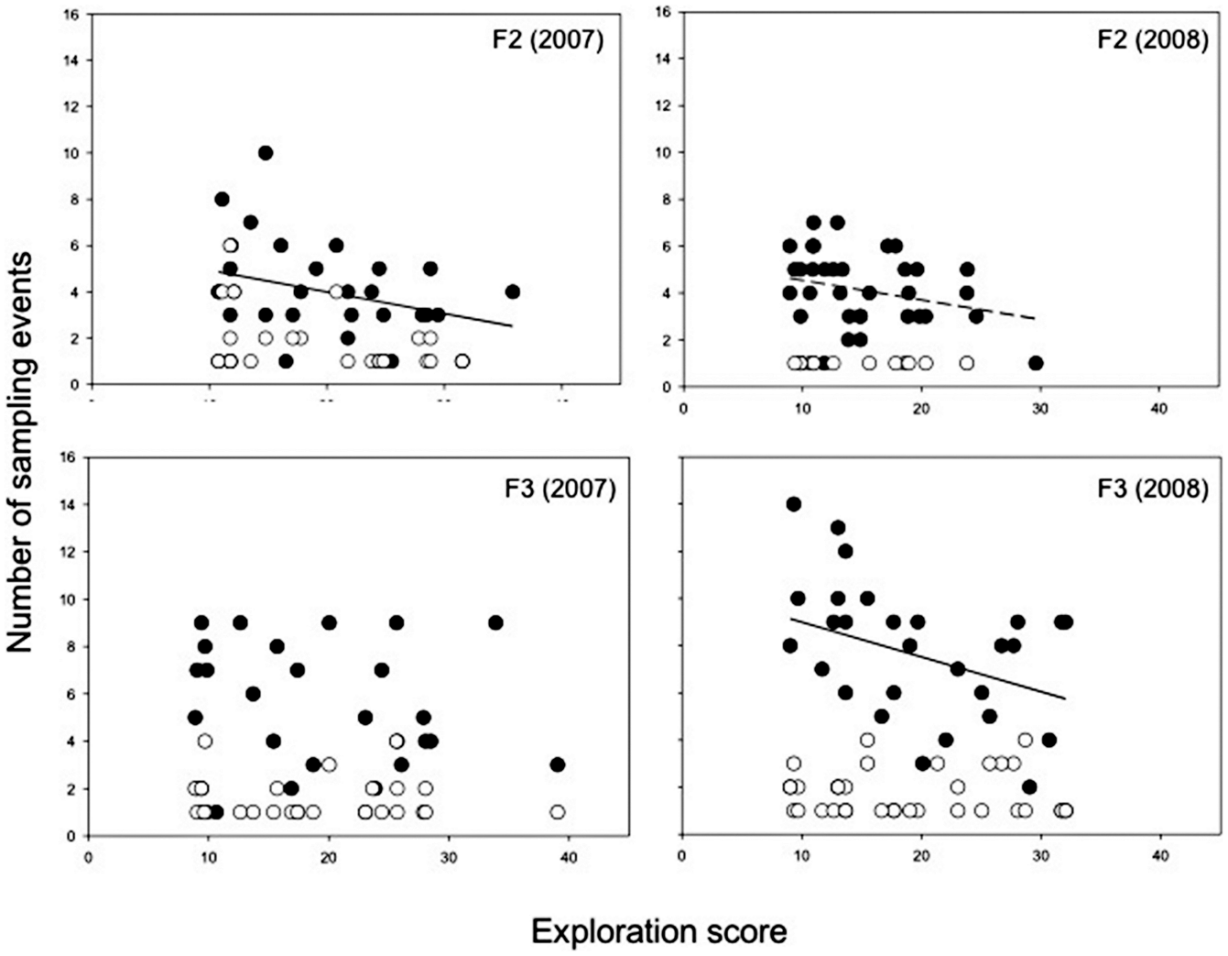
Relationships between exploratory behaviour and number of sampling events at different feeders in summer. Number of sampling events at two experimental feeders (F2 and F3) after food removal plotted against exploratory behaviour, over two summers (2007 and 2008). Closed dots are the number of sampling events on the first day of food removal and open dots the number of sampling events after the first day (day 2–5). The lines are fitted regressions lines for the first day of food removal (dashed line: p<0.15, solid line: p<0.05). Note that for the analyses data on different feeders were pooled and that the overall relationship between exploration score and sampling rate was negative (p = 0.0004), but changed with days since food removal (p = 0.015).

In summer, the visiting rate before food removal was on average 11.9 visits/day ±0.16 s.e. (range 1–27, n = 134 individuals). Visiting rates differed among feeding stations between years (GLMM, interaction year*sites F_1, 145_ = 31.5, p = 0.001, β ± s.e.  = −6.1±1.1) and decreased with date (F_1, 751_ = 6.62, p = 0.01, β ± s.e.  = −0.16±0.06). Visiting rates differed consistently among individuals (LRT: χ^2^ = 78.46, p<.0001), but did not vary with respect to sex, dispersal status, body condition or exploration score (all p>0.2). After the removal of food the average sampling rate was 3.7 sample events/day ±0.19 s.e. (range 1–14 during 1–4 days, n = 124 individuals). Sampling occurred primarily on the first day of food removal (figure 2) and strongly decreased afterwards (p<.0001, table 3). Sampling rates differed strongly among feeding stations both within and between years (i.e. similar pattern as with visiting rates, Table 3). Taking into account the effects of year and site, we found that exploratory behaviour correlated negatively with overall sampling rates (Table 2; see Figure 3 for relationships at different feeders in each year). The negative relationship between exploratory behaviour and sampling rate was especially pronounced on the first day of food removal (F_1, 103_ = 10.36, p = 0.002, β ± s.e.  = −0.11±0.03) and showed an interaction with days since food removal (p = 0.019, table 3, figure 3), indicating that the relationship between exploratory behaviour and sampling rate changed over time. Including pre-manipulation visiting rates in the analyses did not change the results (Table 3). Sampling rates were unrelated to sex, body condition or dispersal status (no significant two-way interactions between exploratory behaviour and other individual characteristics, Table 3). Birds that did not sample the empty feeders (7%) were typically birds that visited the feeder very infrequently before the food removal (F_1, 326_ = 19.69, β ± s.e.  = −6.6±1.49, p<.001) and did not differ from birds sampling empty feeders with respect to any of the individual characteristics (p>0.3).

**Table 3:** Mixed Models on relationship between the number of sampling events after food removal and individual characteristics in a wild population of great tits.

	Summer			Winter		
Variables	F-statistics	*p*	ß ± SE	F-statistics	*p*	ß ± SE
Exploration score	F_1,163_ = 11.55	0.001	−0.10±0.03	F_1,66.7_ = 0.20	0.65	0.003±0.008
visits per day	F_1,90.2_ = 20.26	<.0001	0.23±0.05	F_1,66.6_ = 29.47	<.0001	0.09±0.02
Sex^1^	F_1,91.8_ = 1.04	0.31	−0.29±0.32	F_1,55.3_ = 4.84	0.032	−0.23±0.11
Age^2^				F_1,69.1_ = 2.68	0.11	0.21±0.13
Status^3^	F_1,86.3_ = 1.13	0.29	−0.34±0.32	F_1,55.8_ = 1.12	0.29	−0.11±0.13
Body condition	F_1,90.5_ = 1.09	0.29	0.19±0.18	F_1,66.3_ = 0.10	0.74	0.03±0.08
Days^4^	F_1,85.3_ = 48.39	<.0001	−5.55±0.79	F_1,95.1_ = 59.90	<.0001	−0.61±0.08
Capture method^5^				F_1,62.6_ = 0.17	0.67	−0.05±0.12
Site	F_1,81.7_ = 20.56	<.0001	−3.12±0.43	F_2,72.6_ = 7.83	0.001	
Year	F_1,87.7_ = 3.97	0.049	−2.25±0.44			
Site * Year	F_1,88.2_ = 27.80	<.0001				
Corrected*days^4^	F_1,86.6_ = 6.81	0.011	0.10±0.04			

Random effects included in both models were ID (summer LRT: χ^2^ = 0.15, p = 0.69; winter LRT: χ^2^ = 2.0, p = 0.15). Note that sampling rates in winter were log-transformed to reach normality of residuals.

^1^ Males set to 0.

^2^ Juveniles set to 0.

^3^ Locally born birds set to 0.

4 Days since the removal of food.

5 Roosting in nestbox set to 0.

#### Winter

In winter, the average visiting rate before food removal was 7.8 visits/day ±0.17 s.e. (range 1–23, n = 101 individuals). Visiting rates differed among feeding stations (F_1, 356_ = 11,22, p<.0001) and decreased with date (F_1, 456_ = 41,05, p<.0001, β ± s.e.  = −0.28±0.04). Visiting rates differed consistently among individuals (LRT χ^2^ = 267.94, p<.0001), but did not vary with sex, age, body condition, dispersal status or exploration score (all p>0.1). After food removal, the average sampling rate was 2.8 sample events/day ±0.21 s.e. times (range 1–11 during 1–4 days, n = 80 individuals). Again, sampling occurred primarily on the first day of food removal (figure 2) and strongly decreased afterwards (p<.001, table 3). Sampling rates also differed among feeding stations (p = 0.05, table 3). Sampling rates were lower for males (p = 0.025, table 3), but were unrelated to exploration score, age, body condition or dispersal status (no significant two-way interactions between any of the variables, table 3). Including pre-manipulation visiting rates in the analyses did not change the results (Table 3). Birds that did not sample the empty feeders (20%) were typically birds that visited the feeders very infrequently before the food removal (F_1, 109_ = 25.04, β ± s.e.  = −4.32±0.86, p<.001) and did not differ from birds sampling empty feeders with respect to any of the individual characteristics (p>0.4).

### Rediscovery of food

The rate at which birds rediscovered the food after re-baiting the feeders varied between the three experimental periods (Table 4). Birds that sampled empty feeders more often were more likely to rediscover food in both summer and winter (Table 4), but we found no evidence for an association between the rediscovery of food and exploration score in either summer or winter. Removing sampling events from the model did not result in a significant relationship between exploration score and the rediscovery rate of re-baited feeders (summer, p = 0.96; winter p = 0.45). In both summer and winter rediscovery rates were higher for adult birds (Table 4). Rediscovery rates did not depend on sex, dispersal status or body condition (Table 4).

**Table 4:** Factors affecting the rediscovery of food by great tits after re-baiting the feeding stations, from a Cox proportional hazard model.

	Summer				Winter			
Variables	χ^2^	*p*	ß ± SE	Hazard ratio	χ^2^	*p*	ß ± SE	Hazard ratio
Exploration score	0.27	0.59	0.01±0.02	1.01	1.16	0.28	−0.016±0.015	0.98
Sampling behaviour	4.45	0.035	0.10±0.04	1.10	9.91	0.002	0.13±0.04	1.14
Sex^1^	0.01	0.89	0.03±0.32	1.03	1.41	0.23	0.31±0.26	1.37
Age^2^					9.17	0.003	−0.91±0.30	0.40
Status^3^	1.31	0.25	0.34±0.29	1.40	0.03	0.87	0.04±0.23	1.05
Body condition	0.10	0.75	0.05±0.15	1.05	1.02	0.31	0.19±0.19	1.21
Site	2.05	0.15	0.50±0.35	1.65				
Year	2.03	0.15	−0.44±0.33	0.64				

^1^ Males set to 0.

^2^ Juveniles set to 0.

^3^ Locally born birds set to 0.

## Discussion

When confronted with the sudden disappearance of a previously reliable food source, most great tits in our experiment returned repeatedly to the empty feeders. We interpret this as sampling behaviour whereby birds update their information on the food situation before deciding whether or not to abandon the feeding area. In the two summer experiments, we found that fast-exploring juveniles sampled empty feeders less often compared to slow-exploring juveniles. In winter, however, sampling behaviour was sex-dependent but not related to personality. In both seasons, we found that birds who sampled empty feeders more often were more likely to rediscover food after we again re-baited the feeding stations, but there was no effect of personality.

The negative correlation between exploration behaviour and sampling rates in first-summer juveniles supports our general hypothesis that slow explorers collect more detailed information available in their environment compared to fast explorers. This is consistent with the idea that exploratory behaviour measured in a novel environment reflects differences in information gathering ranging from slow, but thorough, to fast, but superficial exploration [24], [25]. These results thereby provide further evidence that exploratory behaviour measured in captivity reflects relevant differences in foraging behaviour between individuals in the wild [16].

A cautionary note is that differences in sampling rate may actually reflect differences in foraging routines, rather than differences in information gathering. However, this would imply that slow explorers rely more on routines compared to fast explorers in their foraging behaviour, which is contrary to previous observations in the laboratory [24], [25]. We note that observations by Verbeek [25] and Drent & Marchetti [24] were based on very brief (5 min.) observations in small aviaries in which birds had few alternative foraging options, and therefore difficult to compare with the present study. More generally, this interpretation would contradict the overall view of slow explorers having a more reactive coping style [3]. Another explanation might be that slow explorers rely more strongly on artificial feeders as a source of food. However, if this was the case, we would have expected to find differences between fast and slow explorers in visiting rates when feeders were filled, but this was not true. In addition, there is no clear reason why slow explorers would rely more on artificial food, because they are not necessarily lower-ranked in dominance [37] and exploration scores are not directly related to performance or fitness measures [10]. We therefore conclude that the higher sampling rates by slow explorers reflect the continuous exploration of all locally available resources. We acknowledge, however, that more detailed information on the actual intake rates of natural and artificial food are necessary to fully support this hypothesis.

Our results described here add further detail to the previously reported observations from the same experiments in summer, in which we found that fast explorers responded to food removal by rapid shifts to new foraging areas (from day 2 onwards), whereas slow explorers did this more gradually and on a smaller spatial scale [14]. The observed differences in sampling rates between fast and slow explorers may therefore reflect different decision-making rules about when to leave the area of the feeder whereby slow explorers seem to take a more informed decision (i.e. based on more detailed information about their environment and/or available food resources) compared to fast explorers. These findings show similarities with the cue dependency of coping strategies described in rodents, whereby reactive individual tend to rely more on feedback information, while proactive individuals act more on the basis of previous experience (i.e. internally driven predictions), which may be fast, but inaccurate [2], [19], [21].

The finding that personality differences in sampling behaviour were expressed in first-summer juveniles, but not in first-winter juveniles and adults, suggests that such personality effects may only be visible in birds with limited environmental knowledge and/or foraging skills, given that juveniles in summer only recently reached independence. Alternatively, the lack of a relationship between sampling rates and exploration score in winter may also be explained by general differences in food availability. For example, the relationships in winter may have been masked by individual variation in knowledge on alternative food resources unrelated to exploration behaviour; given the presence of many other artificial food resources in people's gardens surrounding the experimental sites, this effect may have been more pronounced in winter than summer. We have no clear explanation why in winter females sampled empty feeders more often compared to males, but one possibility might be that this reflects local dominance effects [38], [39]. For example, the presence of local territorial males around other feeders may force subdominant females to explore a wider range of other available resources. Such effects are likely to be much stronger in the months before breeding compared to summer [40] and are not necessarily linked to variation in personality [37].

The finding that sampling rates were positively related to the rate of rediscovering food confirmed our hypothesis that sampling behaviour may indeed confer adaptive benefits and be an important component underlying behavioural flexibility. Contrary to our expectations, however, and despite a correlation between exploration and sampling behaviour in first-summer juveniles, no link was found between exploration behaviour and the rate of rediscovering refilled feeders. The adaptive value of a higher investment in information gathering trough sampling by slow explorers therefore remains unclear. However, a number of factors might have confounded associations between exploratory behaviour and the rediscovery of food. Firstly, it is possible that fast explorers collected information on empty feeders without entering the cage and being recorded, for instance, by using social cues such as the presence or absence of other birds around the feeder. This would still imply that fast and slow explorers differed in their sampling method, but with limited consequences on the information obtained. We believe this explanation to be unlikely, because (i) exploratory behaviour was unrelated to time spent sampling (p>0.4 in both summer and winter; details not shown) and (ii) data on a subset of 29 birds fitted with both a pit-tag and a radio-tag confirmed that sampling rate reflected the time spent in the vicinity of the feeder (correlation with the average distance to the feeder on the day of the manipulation: Pearson correlation, r = −0.43, p = 0.02). Secondly, juveniles in summer forage in unstable flocks [41] and depending on the composition of these flocks fast explorers may have been directed to the feeders by slow explorers (i.e. producer-scrounger effects [42], but see [43], [44] for an opposite pattern). Finally, the fact that older birds in winter were more likely to rediscover food shows that foraging experience and local familiarity with the environment may also play an important role in explaining differences in behavioural flexibility. Hence, knowledge on the exact history of birds (i.e. date of arrival at feeders and previous foraging sites) may be required to fully evaluate the adaptive value of personality differences in information gathering tactics.

To conclude, our study shows that fast and slow explorers differ in their sampling strategy when confronted with a sudden change in food conditions. These results are consistent with the view of personalities as different styles of coping with environmental changes and fit the recent notion that personality types may exhibit alternative strategies for managing uncertainty [20], [45]. However, the observed association between personality and sampling rates were rather subtle and unrelated to the rediscovery of food after refilling the feeders. The adaptive value of these alternative behavioural tactics therefore needs to be further explored.
